# Wideband Tympanometry Findings in Otitis Media with Effusion—A Systematic Review

**DOI:** 10.3390/jcm15114255

**Published:** 2026-05-31

**Authors:** Jakub Osowski, Bogusław Mikaszewski, Tomasz Przewoźny, Aleksandra Klein-Obrębska

**Affiliations:** 1 Department of Otolaryngology, Medical University of Gdańsk, Mariana Smoluchowskiego 17, 80-214 Gdańsk, Poland; boguslaw.mikaszewski@gumed.edu.pl (B.M.); tomasz.przewozny@gumed.edu.pl (T.P.); aleksandra.klein@gumed.edu.pl (A.K.-O.); 2 Department of Otolaryngology, University Clinical Center, 80-952 Gdańsk, Poland; 3 First Doctoral School, Medical University of Gdańsk, 80-309 Gdańsk, Poland

**Keywords:** otology, audiology, otitis media, middle ear, tympanometry, tympanic effusion, wideband tympanometry

## Abstract

**Background/Objective:** Otitis media with effusion (OME) is a prevalent condition in the pediatric population worldwide. If left untreated, it can lead to serious complications such as permanent middle ear damage and hearing loss. **Methods**: This study aims to evaluate the utility of wideband tympanometry (WBT) in diagnosing OME in children. A comprehensive literature search was conducted independently by two authors across three major databases—PubMed, Scopus, and Embase covering the period from 1 January 2000 to 11 November 2025. Discrepancies in data extraction or interpretation were resolved by consensus. **Results**: The study analyzed data from 1115 ears with surgically confirmed tympanic effusion and compared these with 721 ears from healthy controls. The most significant reduction in absorbance values was observed in the low- and mid-frequency ranges. These absorbance parameters effectively differentiated WBT results obtained in healthy children from those in patients with surgically confirmed effusion during myringotomy. **Conclusions:** In ears with effusion, characteristic alterations in absorbance patterns—reflecting increased stiffness of the middle ear’s mechanical system—can be identified via wideband tympanometry. This novel audiological assessment shows promise for the diagnosis of OME; further research is warranted to validate its clinical utility and to compare it with existing diagnostic methods.

## 1. Introduction

Otitis media with effusion (OME) is a prevalent condition in the pediatric population, affecting approximately 50–90% of children under 5 years of age [1,2]. The condition typically progresses insidiously and is often asymptomatic, with no acute or obvious symptoms. A hallmark feature is the presence of serous or mucoid effusion within the middle ear cavity, leading to conductive hearing loss [3]. Diagnosis primarily relies on clinical presentation (notably hearing impairment), otoscopic examination findings, assessment of tympanic membrane mobility via pneumatic otoscopy, and standard single-tone tympanometry [1,3].

The middle ear can be conceptualized as a mechanical resonator system. This analogy was first described by the German physician Hermann von Helmholtz in 1868. Helmholtz detailed the mechanics of the tympanic membrane and ossicular chain [4,5], and introduced the concept of the middle ear functioning as a resonator comprising two main components: the resonator cavity and the narrow neck connecting to the external environment. Resonance within this system depends on the elasticity (or compliance) of the air contained within the cavity and the mass of air within the narrow neck [6,7]. The system resonates at a specific, characteristic frequency determined by the vibratory behavior of the air mass in the neck and the elastic properties of the cavity. Alterations in the air mass or the elasticity of the cavity modify the resonance frequency [8,9].

Wideband tympanometry (WBT) evaluates the transmission of mechanical sound waves through middle ear structures across a broad frequency spectrum (0.250–8 kHz), expressed as “absorbance.” An absorbance of 0% indicates no sound transmission, while 100% signifies complete transmission of sound energy to the inner ear. This relatively new testing modality is increasingly used in routine audiological practice [10,11], with two commercially available systems: Titan (Interacoustics) and Mimosa Acoustics [11]. Unlike conventional tympanometry, WBT measures absorbance at both the tympanometric peak pressure (TPP) and ambient pressure, facilitating assessments even after procedures such as tympanoplasty, stapedotomy, or in cases of tympanic membrane perforation or patent ventilation tubes, which allows for thorough middle ear diagnostics in broad spectrum of diseases [12,13,14,15].

To explore these theoretical principles and validate them clinically, the authors conducted a systematic review to identify specific WBT (wideband tympanometry) alterations observed in pediatric patients with OME and to test the research hypothesis that increased stiffness of the middle ear, secondary to effusion, results in decreased absorbance at low and mid frequencies.

## 2. Materials and Methods

### 2.1. Search Strategy

To evaluate the utility of wideband tympanometry (WBT) in the pediatric population with otitis media with effusion (OME), a systematic review was conducted in accordance with PRISMA (Preferred Reporting Items for Systematic Reviews and Meta-Analyses) guidelines [16]. PRISMA Checklist is available as a Supplementary Materials. The review has not been registered. Comprehensive searches were performed across three major bibliographic databases: PubMed (National Library of Medicine), Scopus (Elsevier), and Embase (Elsevier). The search covered the period from 1 January 2000 to 11 November 2025.

### 2.2. Searching and Data Screening

**Figure 1:**
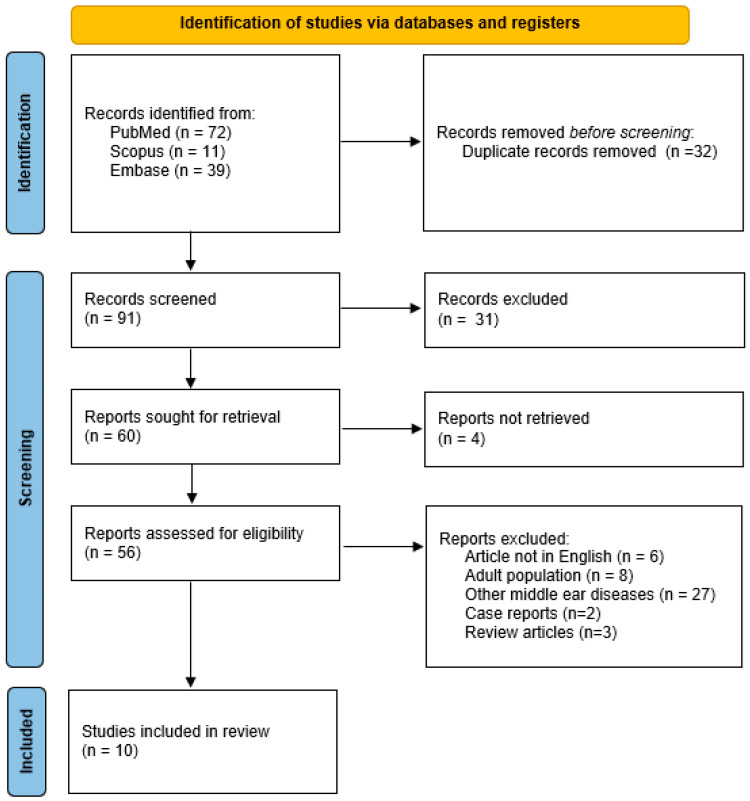
PRISMA flowchart.

On 20 November 2025, two independent authors (J.O. and B.M.) conducted searches of the aforementioned databases. Due to the differences in used terminology, the search formula was complex and included various possible terms commonly used in the literature to describe wideband tympanometry and otitis media with effusion. Exact search formula: (‘wideband tympanometry’ OR ‘wideband absorbance’ OR ‘wideband immitance’ OR ‘absorbance pattern’) AND (‘otitis media with effusion’ OR ‘tympanic effusion’ OR ‘effusion’ OR ‘secretory otitis media’ OR ‘otitis media’). The initial search identified 122 articles, of which 32 duplicates were removed. Remaining 91 records underwent title and abstract screening, resulting in the exclusion of 31 articles primarily due to the absence of WBT methodology description. The remaining 60 articles were subjected to full-text review, of which 10 met all inclusion criteria and were incorporated into this systematic review. The screening process is depicted in the PRISMA flow diagram (Figure 1). Discrepancies between reviewers were resolved through consensus.

### 2.3. Eligibility Criteria

This systematic review adhered to a structured PICOTS framework. Population: Pediatric patients with OME scheduled for elective myringotomy. Intervention: Audiological assessment incorporating WBT. Comparison: Healthy pediatric controls without middle ear pathology not undergoing surgical intervention. Outcome: WBT parameters demonstrating significant differences between groups. Timing and Setting: Routine diagnostic assessments performed preoperatively prior to elective myringotomy. Exclusion criteria encompassed adult populations, other chronic middle ear diseases that could influence audiometric outcomes, genetic ear and hearing disorders, case reports, literature reviews, and publications not in English (Table 1).

### 2.4. Data Extraction and Processing

Given the limited number of eligible studies, data extraction was performed manually by the same two reviewers involved in the search process. Extracted data included: (1) study details (authors, publication year), (2) sample sizes of the control group (healthy individuals) and the OME group, (3) statistically significant differences in absorbance at specific frequencies, mean absorbance values, and resonance frequency, and (4) cutoff thresholds for relevant parameters when reported. The methodological quality and risk of bias in each study were assessed using the Newcastle–Ottawa Scale [17].

## 3. Results

### 3.1. Database Search Results

Initial manual screening of the listed databases yielded 122 records, which was reduced to 91 after removing duplicates. Following title and abstract screening, 31 articles were excluded for not meeting the PICOTS criteria [18]. Subsequently, 60 articles qualified for full-text review, but four could not be retrieved. Of the remaining 56 articles, 46 were excluded for various reasons: six articles were in languages other than English (Japanese and Chinese), eight studies involved adult populations, 27 papers described WBT application in conditions other than otitis media with effusion (OME), three were review articles, and two were case reports. Ultimately, 10 articles met the inclusion criteria for this systematic review.

### 3.2. Included Study Characteristics

In a study by Senturk et al. [19], patients undergoing myringotomy and healthy controls were compared based on parameters obtained during wideband tympanometry (WBT), including resonance frequency, compliance at 226 Hz, absorbance, and tympanic peak pressure (TPP). The study identified statistically significant differences between the groups that allowed detection of middle ear effusion but did not reliably differentiate effusion viscosity (mucoid vs. serous).

Callahan et al. [20] conducted a prospective observational study on children scheduled for elective myringotomy due to suspected OME. The study lacked a healthy control group. Absorbance measurements in the low-frequency range (577–1259 Hz) effectively differentiated between patients with surgically confirmed effusion and those without effusion (i.e., normal ear at myringotomy). Unlike the Senturk study, significant differences in absorbance were observed between different effusion types: purulent effusion exhibited the lowest absorbance, mucous effusion showed intermediate levels, and serous effusion approached normal values.

Elbattat et al. [21] compared WBT parameters (resonance frequency, TPP, and absorbance) between healthy children and those with tympanic effusion. The effusion group showed statistically significant reductions across all parameters, with the most pronounced decrease in low-frequency absorbance (250, 500, and 1000 Hz).

Terzi et al. [22] performed a similar comparison between healthy children and those with suspected OME undergoing myringotomy. In the effusion group, absorbance was decreased across all frequencies, notably in the low-frequency range (0.375–2 kHz). In children without confirmed effusion, absorbance values closely resembled those of healthy controls.

Liang et al. [23] evaluated absorbance at two pressure conditions—pressure of the greatest compliance (TPP) and ambient pressure. Patients with middle ear effusion demonstrated reduced absorbance at both pressures, although differences were not statistically significant. The most notable differences were in the 0.47–1.03 kHz frequency range.

Sanford and Brockett [24] assessed absorbance in children with various middle ear pathologies, including OME, comparing results with healthy controls. Children with effusion showed reduced absorbance across frequencies, with the greatest reduction in the 1000–2000 Hz range.

Yildiz et al. [25] studied the natural progression of OME over three months, with assessments at baseline, 1, 2, and 3 months. WBT, standard tympanometry, and pneumatic otoscopy were performed. The most significant differences compared to healthy controls were observed at 2000 Hz during initial assessment.

Ellison et al. [26] compared healthy children with those scheduled for myringotomy due to OME, using pneumatic otoscopy and WBT. The OME group exhibited decreased absorbance across all frequencies, with the most significant changes between 1.5 and 3 kHz.

Aithal et al. [27] conducted a similar comparative study, analyzing absorbance at TPP and ambient pressure. No significant differences between pressure conditions were observed. Both groups showed globally reduced absorbance, most notably around 1000 Hz.

Merchant et al. [28] compared healthy children with those scheduled for myringotomy for suspected OME to assess the relationship between effusion volume and WBT results. The effusion group demonstrated decreased absorbance across nearly the entire frequency spectrum, with peak significance between 1 and 5 kHz. A correlation was observed between the amount of effusion and absorbance reduction; ears with full effusion showed greater absorbance decrease than partially effused ears.

### 3.3. Risk of Bias Assessment

All included studies were evaluated using the Newcastle–Ottawa Scale, demonstrating low risk of bias (scores ranging from 7 to 9). Potential biases include the absence of a matched healthy pediatric comparison group in the studies by Callahan et al. and Yildiz et al., as well as variability in tympanometric equipment. Two earlier studies (Ellison et al. [26] and Aithal et al. [27]) utilized prototype systems from Interacoustics, whereas Senturk et al. did not specify their equipment. The remaining studies employed commercially available Titan software (Interacoustics).

### 3.4. Results of Data Synthesis

Combining data from all included studies, a total of 1115 ears with surgically confirmed middle ear effusion and 721 ears serving as controls were analyzed. All studies consistently reported decreased mean absorbance across all frequencies in ears with effusion. While the specific frequencies with the most statistically significant reductions varied slightly between studies, the most consistent findings were in the low- to mid-frequency range (250–2000 Hz). Additional parameters, such as the relationship between TPP and ambient pressure [19,23,27] and resonance frequency [19,21], were evaluated in fewer studies. A detailed summary of the findings is provided in Table 2 and Table 3.

## 4. Discussion

In the pediatric population, otitis media with effusion (OME) most commonly occurs in children under 5 years of age [1,2,3]. This is a critical period for speech and communication development. Persistent effusion within the middle ear (tympanic cavities) impairs the mechanical transmission of sound waves, leading to conductive hearing loss that can negatively affect auditory perception, speech development, vocabulary acquisition, and higher-level social skills. Such children may experience academic difficulties [2]. Additionally, fluctuating and asymmetric conductive hearing loss associated with OME can disrupt the development of auditory processing centers in the brain, potentially resulting in binaural hearing impairments [29]. In the case of monoaural hearing impairment, speech development will proceed normally, but spatial hearing will not develop properly; the child will not be able to localize the source of sound and it will also be difficult to understand speech in noisy environments [3]. Other complications include mechanical damage to middle ear structures (auditory ossicles, tympanic membrane), which can result in chronic otitis media and permanent hearing impairment.

Given these risks, prompt diagnosis and management of OME are essential. Diagnosis is primarily achieved through otoscopy, pneumatic otoscopy, and tympanometry with a 226 Hz probe tone [2,3,30]. However, misdiagnosis can occur, sometimes leading to unnecessary myringotomies [31]. Takata et al. [32] conducted a systematic review and meta-analysis of diagnostic methods used from 1966 to 2000, indicating that pneumatic otoscopy offers the highest specificity and sensitivity, though its accuracy is dependent on the experience of the examiner [33]. Standard tympanometry is less reliable, and at the time of that review, wideband tympanometry (WBT) was not yet available. In a study by Sundgaard et al. [34], 1409 pediatric patients—including those with OME, acute otitis media, and healthy controls—were examined by otolaryngologists and independently assessed via otoscopic images by other ENT specialists blinded to clinical data. Diagnosing acute otitis media was relatively straightforward, but diagnosing OME based solely on otoscopy was more challenging. Incorporating tympanometric data, particularly WBT, increased diagnostic accuracy. These findings highlight the difficulty in detecting tympanic effusion and suggest that combining multiple diagnostic modalities improves accuracy; further research is needed in this area.

As mentioned earlier, there are two main WBT platforms available. Most of the studies included in this review used commercially available WBT system (Titan by Interacoustics, Denmark). Those papers (Callahan [20], Elbattat [21], Terzi [22], Liang [23], Sanford [24], Yildiz [25], and Merchant [28]) have provided similar results, in ears with effusion the mean absorbance was decreased, particularly in the low and mid frequencies. However, the earlier prototype WBT system by Interacoustics (which was used in Ellison [26] and Aithal [27] studies) yielded similar results, regarding the most important frequency range. These two systems by Interacoustics differ primarily in the presentation of results (the prototype provided only raw numerical values, while the commercially available system also generates results in the form of graphs, which are easier to read and interpret—similar to standard single-tone tympanometric equipment). The manufacturer’s calibration procedure is the same, but some differences can always arise during technical inspections and recalibration by local service centers. Study by Senturk [19] did not provide clear data about used equipment. None of the research studies used OtoStat system by Mimosa Acoustics (USA).

Resonance frequency (RF) measured during WBT is another parameter of interest; it is based on the Helmholtz resonator model [35]. Kourelis et al. [36] found that RF was elevated in ears with surgically confirmed effusion, and this increase was statistically significant, correlating with adenoid hypertrophy confirmed endoscopically in patients with Type A tympanograms and normal otoscopic findings. Conversely, Senturk et al. [19] and Elbattat et al. [21] observed decreased RF in effused ears, contrary to the theoretical expectation of increased RF, indicating possible discrepancies. Both studies noted decreased absorbance at low frequencies (500–1000–2000 Hz), consistent with increased stiffness of middle ear transmission. These conflicting results underscore the need for further investigation into RF alterations in middle ear effusion.

Differentiating effusion characteristics using WBT remains an unresolved issue requiring further study. Callahan et al. [20] classified ears undergoing myringotomy into four groups: empty, serous, mucous, and purulent effusions. Purulent effusions showed the greatest reduction in absorbance, with mucous effusions exhibiting a moderate decrease. Distinguishing between ears without effusion and those with serous effusion can be challenging, as both may have near-normal absorbance values—a phenomenon possibly related to differences in fluid density, with serous fluid being less stiffening than mucous [37]. Senturk et al. [19] did not find statistically significant differences between serous and mucous effusions. Merchant et al. [28] categorized effusions based on subjective volume assessment during myringotomy, observing the greatest absorbance reduction in ears with full effusions. Overall, current evidence suggests that while WBT can detect the presence of effusion, differentiating its specific type remains complex and warrants further research.

## 5. Conclusions

This systematic review highlights the utility of wideband tympanometry (WBT) in the diagnosis of otitis media with effusion (OME) in the pediatric population. The most characteristic audiometric alterations in ears with effusion include a reduction in absorbance across the entire frequency spectrum, with the most pronounced decrease observed in the low and mid-frequency ranges (500–2000 Hz). This pattern is attributable to increased middle ear stiffness, consistent with the Helmholtz resonator model. However, due to the relatively small number of studies included in the review (10) and their heterogeneity (different assessment protocols, different types of equipment used for audiometric testing), drawing clear and conclusive conclusions is not entirely possible. Further research is warranted to compare WBT with existing diagnostic modalities for OME and to perform meta-analyses synthesizing current data on absorbance values and resonance frequency. Such efforts are essential to establish precise numerical cutoff thresholds, thereby facilitating the integration of WBT into standard diagnostic protocols for OME.

## Figures and Tables

**Table 1:** Inclusion and exclusion criteria.

Inclusion Criteria	Exclusion Criteria
Children population [age 2–16 years] OME scheduled for elective myringotomy WBT testing done prior to surgery Year of publication 2000–2025	Adult population [age > 16 years] Other diseases of middle ear Genetic disorders affecting ear/hearing Language of study other than English Case reports/reviews
Studies in English	

**Table 2:** Data extracted from the articles.

First Author Name	Comparison Group [Ears] *	Study Group [Ears]	Newcastle–Ottawa Score	Used Equipment
Senturk et al. [19]	160	244	9	No information
Callahan et al. [20]	N/A	211	7	Titan, Interacoustics
Elbattat et al. [21]	100	176	9	Titan, Interacoustics
Terzi et al. [22]	60	112	8	Titan, Interacoustics
Liang et al. [23]	182	136	8	Titan, Interacoustics
Sanford et al. [24]	92	20	7	Titan, Interacoustics
Yildiz et al. [25]	N/A	48	8	Titan, Interacoustics
Ellison et al. [26]	53	59	8	Prototype system, Interacoustics
Aithal et al. [27]	60	60	7	Prototype system, Interacoustics
Merchant et al. [28]	14	49	9	Titan, Interacoustics

* N/A—not available.

**Table 3:** Data extracted from the articles (continued).

First Author Name	Resonance Frequency	Transtympanic Pressure	Average Absorbantion Across All Frequencies	Most Statistically Significant Absorbance Ranges [Hz]
Senturk et al. [19]	Decreased	No correlation found	Decreased	500, 1000, 2000
Callahan et al. [20]	Not examined	Not examined	Decreased	577–1259
Elbattat et al. [21]	Decreased	Decreased	Decreased	250, 500, 1000
Terzi et al. [22]	Not examined	Not examined	Decreased	375–2000
Liang et al. [23]	Not examined	Not examined	Decreased	470–1030
Sanford et al. [24]	Not examined	Not examined	Decreased	1000–5000
Yildiz et al. [25]	Not examined	Not examined	Decreased	2000
Ellison et al. [26]	Not examined	Not examined	Decreased	1500–3000
Aithal et al. [27]	Not examined	Not examined	Decreased	1250–1500
Merchant et al. [28]	Not examined	Not examined	Decreased	1000–5000

## Data Availability

The raw data supporting the conclusions of this article will be made available by the authors without undue reservation.

## Supplementary Materials

The following supporting information can be downloaded at: https://www.mdpi.com/article/10.3390/jcm15114255/s1. PRISMA 2020 Checklist.

## Abbreviations

The following abbreviations are used in this manuscript:

TPP	tympanometric peak pressure
WBT	wideband tympanometry
RF	resonance frequency
OME	otitis media with effusion
